# Contrasting the relationship of serum uric acid/albumin ratio on quantitative flow ratio with other multiple composite parameters in patients with suspected coronary artery disease

**DOI:** 10.1186/s12872-024-03763-9

**Published:** 2024-03-05

**Authors:** Jin Liu, Han Wei, Xuanzhi Zhu, Huangjun Liu, Lijun Jin

**Affiliations:** 1 Department of Cardiology, The First Affiliated Hospital of Yangtze University, No.8, Aviation Road, Shashi District, Jingzhou, 434021 China; 2 Department of Anesthesiology, Huanggang Central Hospital of Yangtze University, Huanggang, China

**Keywords:** Coronary artery disease, Quantitative flow ratio, Serum uric acid, Albumin, Logistic regression

## Abstract

**Objective:**

The aim of this study was to investigate the association between quantitative coronary flow reserve (CFR) and the blood uric acid/albumin ratio, as well as multiple clinical parameters, in order to assess the severity of coronary artery functional stenosis.

**Methods:**

This retrospective cross-sectional study included 257 suspected coronary artery disease patients who underwent coronary angiography (CAG) and quantitative flow ratio (QFR) examinations in the Department of Cardiovascular Medicine at the First Affiliated Hospital of Yangtze University in Jingzhou City, China, between September 2022 and March 2023. The study subjects were divided into two groups based on their QFR values: QFR ≤ 0.80 group and QFR > 0.80 group. Correlation of uric acid-to-albumin ratio (UAR), high-density lipoprotein ratio (MHR), systemic immune-inflammation index (SII), Systemic Inflammation Response Index (SIRI), and Aggregate Index of Systemic Inflammation (AISI) with coronary artery QFR was analyzed using univariate and multivariate logistic regression models, considering them as both continuous and binary variables.

**Results:**

The QFR ≤ 0.80 group consisted of 83 patients, while the QFR > 0.80 group included 174 patients. Significant differences were observed between the QFR ≤ 0.80 and QFR > 0.80 groups in the following parameters: UAR (9.19 ± 2.47 vs 7.61 ± 1.91; *p* < 0.001), MHR (0.46 ± 0.19 vs 0.37 ± 0.16, *p* < 0.001), SII (674.98 ± 332.30 vs 571.43 ± 255.82; *p* = 0.006), SIRI (1.53 ± 0.83 vs 1.29 ± 1.10; *p* = 0.047), and AISI (340.22 ± 242.10 vs 243.97 ± 151.97; *p* < 0.001). ROC curve analysis revealed an area under the curve of 0.701 (CI: 0.633–0.770; *p* < 0.001) for UAR. In the univariate analysis, when treated as binary variables, high levels of UAR, MHR, SII, SIRI, and AISI were found to be significantly associated with the risk of QFR ≤ 0.80 (all *P* < 0.05). However, in the multivariate regression analysis, only high levels of UAR and AISI remained significantly associated with QFR ≤ 0.80 (all *P* < 0.05). When treated as continuous variables, the univariate analysis indicated that UAR (OR: 1.412, CI: 1.231–1.620, *p* < 0.001), e^MHR (OR: 1.394, CI: 1.151–1.687, *p* < 0.001), lnSII (OR: 1.001, CI: 1.000–1.002, *p* = 0.008), and lnAISI (OR: 2.695, CI: 1.539–4.719, *p* = 0.001) were significantly associated with QFR ≤ 0.80. In the multivariate analysis, UAR (OR: 1.373, CI: 1.187–1.587, *p* < 0.001) and AISI (OR: 2.217, CI: 1.309–3.757, *p* < 0.001) remained significantly associated with QFR ≤ 0.80.

**Conclusions:**

The results of this study indicate a significant association between UAR and AISI with QFR ≤ 0.80, suggesting its potential role in predicting the extent of functional coronary artery stenosis in patients with CAD. Additionally, AIRI, identified as an inflammatory marker in the complete blood count, was found to exert influence on the severity of coronary artery physiology.

## Introduction

Coronary heart disease is a common cardiovascular disease with a rising incidence worldwide. According to the latest research data, it is one of the leading risk factors of heart disease and cardiovascular events [1]. The pathogenesis of coronary heart disease involves multiple aspects. The most common is the atherosclerotic lesion of coronary arteries, characterized by lipid deposition, fibrous plaque formation, and plaque rupture. This leads to narrowing and blockage of the coronary arteries, resulting in myocardial ischemia and angina. In addition, coronary heart disease involves the activation of inflammatory responses. Inflammation plays a significant role in the development of coronary heart disease, including infiltration of inflammatory cells, release of inflammatory mediators, and activation of inflammatory signaling pathways. Inflammatory responses are closely associated not only with plaque formation and rupture but also with endothelial dysfunction, platelet aggregation, and thrombus formation [2–4]. In recent years, many scholars have reported on the role of leukocyte telomeres in atherosclerosis [5–7], and novel indices such as the systemic immune-inflammation index (SII), systemic inflammation response index (SIRI), and comprehensive systemic inflammation index (AISI) have been derived to serve as objective markers for assessing the balance between host systemic inflammation and immune response. These indices have been shown to have predictive value for the prognosis of cardiovascular diseases [8–15]. Some studies have revealed other parameters calculated by combining different biochemical indicators or individual biochemical indicators. For example, the monocyte-to-HDL cholesterol ratio (MHR) has been shown to be associated with mortality in patients with coronary heart disease [16, 17]. The uric acid-to-albumin ratio (UAR) has been found to be related to the prognosis of coronary heart disease [15, 18–20].

Coronary angiography has become increasingly widespread in the assessment of coronary arteries. However, it has limitations as it can only provide a visual estimation of the degree of vessel stenosis and cannot accurately evaluate the presence of myocardial ischemia or reflect the three-dimensional structure and stability of coronary plaques. Intracoronary functional and imaging techniques, such as fractional flow reserve (FFR), intravascular ultrasound (IVUS), and optical coherence tomography (OCT), have ushered in the era of precision treatment in coronary intervention. FFR is recognized as the gold standard for assessing the severity of coronary artery stenosis [1]. However, these intracoronary functional and imaging examinations require high technical expertise and increase the use of surgical materials, leading to potential complications and increased medical costs. In recent years, quantitative flow ratio (QFR), an image-based rapid assessment technique for coronary artery functional evaluation, has gained attention. Numerous studies conducted in China and abroad have confirmed the value of QFR in the assessment of coronary artery physiology, and the FAVOR III China study published in a reputable journal has demonstrated the high health-economic value of QFR [21]. Currently, the comparative value of different blood component parameters in assessing the degree of functional coronary artery stenosis has not been extensively explored. Therefore, we conducted this study to investigate the relationship between these parameters (UAR, MHR, SII, SIRI, and AISI) and coronary artery physiology. The aim of this study is to quantify the degree of coronary artery stenosis using QFR and further explore the relationship between UAR, MHR, SII, SIRI, AISI, and the extent of coronary artery functional abnormalities in patients.

## Materials and methods

### Patient selection

A total of 410 suspected coronary heart disease patients who underwent coronary angiography (CAG) and QFR testing in the Cardiovascular Department of the First People's Hospital of Jingzhou City from September 2022 to March 2023 were collected.

Inclusion criteria: (1) Age 18 years and above; (2) Completion of coronary angiography examination; (3) Presence of clinical symptoms of coronary artery stenosis, such as chest pain, angina, etc.; (4) Quantitative assessment of coronary artery stenosis is required using the QFR technique.

Exclusion criteria: (1) Patients with unsatisfactory coronary angiography images (coronary ostial lesions, severe vessel tortuosity, diffuse long lesions, poor coronary artery image quality, lack of two images with a difference of more than 25 degrees, overlapping target lesions, excessive shrinkage or inadequate filling of contrast agent); (2) Patients with acute coronary syndrome (including acute myocardial infarction and unstable angina), coronary artery occlusion, diagnosed congenital coronary artery anomalies, or myocardial bridge; (3) Patients with severe valve disease, severe heart failure, cardiogenic shock, known history of liver or kidney failure (abnormal liver function defined as chronic liver disease, such as liver fibrosis or bilirubin > 2 times the upper limit of normal or alanine aminotransferase > 3 times the upper limit of normal, abnormal kidney function defined as chronic dialysis or kidney transplantation or glomerular filtration rate < 30 ml/min); (4) Patients with active infection, chronic inflammatory conditions, autoimmune diseases, hematologic disorders, and malignant tumors; (5) Patients with a history of medication use that may affect uric acid levels and those with missing data; (6) Patients with a history of previous coronary artery intervention surgery.

**Fig. 1:**
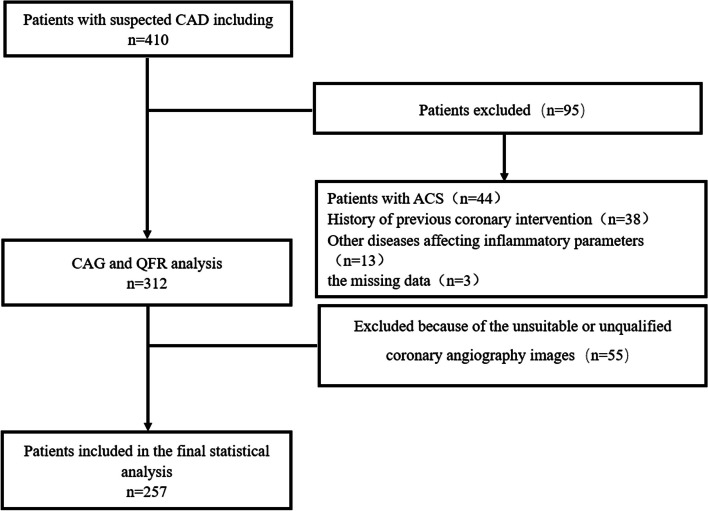
Study flow chart CAD, coronary artery disease; CAG, coronary arteriography; QFR, quantitative flow ratio

Based on the inclusion and exclusion criteria, consecutive coronary heart disease patients who underwent CAG and QFR were enrolled, and a final total of 257 cases were included (Fig. 1). According to the critical value of QFR, the suspected coronary heart disease patients in this study were divided into two groups: QFR ≤ 0.8 (*n* = 83) and QFR > 0.8 (*n* = 174).

This study met the review criteria set by our ethics committee and was approved by the ethics committee. As the study was retrospective in design, the ethics committee exempted the acquisition of informed consent.

### Data collection and detection methods

Population demographic characteristics and laboratory data were extracted from the hospital's inpatient system. The collected demographic characteristics encompassed gender, age, smoking status, medical history, and a history of previous coronary artery intervention. The medical history included conditions such as hypertension, diabetes, stroke, liver and kidney diseases, as well as hematological disorders. The laboratory data comprised comprehensive assessments including routine blood tests, lipid profile, liver function, renal function, and electrolyte levels, all obtained upon admission.

Prior to undergoing CAG, peripheral venous blood samples were meticulously collected from patients who had observed a minimum 8-h fasting period overnight. Standardized methods were employed to meticulously analyze these samples, ensuring the avoidance of any potential storage-related influences. The study incorporated the calculation of five composite parameters as follows: UAR = uric acid/albumin ratio; MHR = monocyte / HDL-cholesterol ratio; SII = neutrophil × platelet/lymphocyte; SIRI = neutrophil × monocyte/lymphocyte; AISI = neutrophil × platelet × monocyte/lymphocyte.

### Calculation of the QFR

**Fig. 2:**
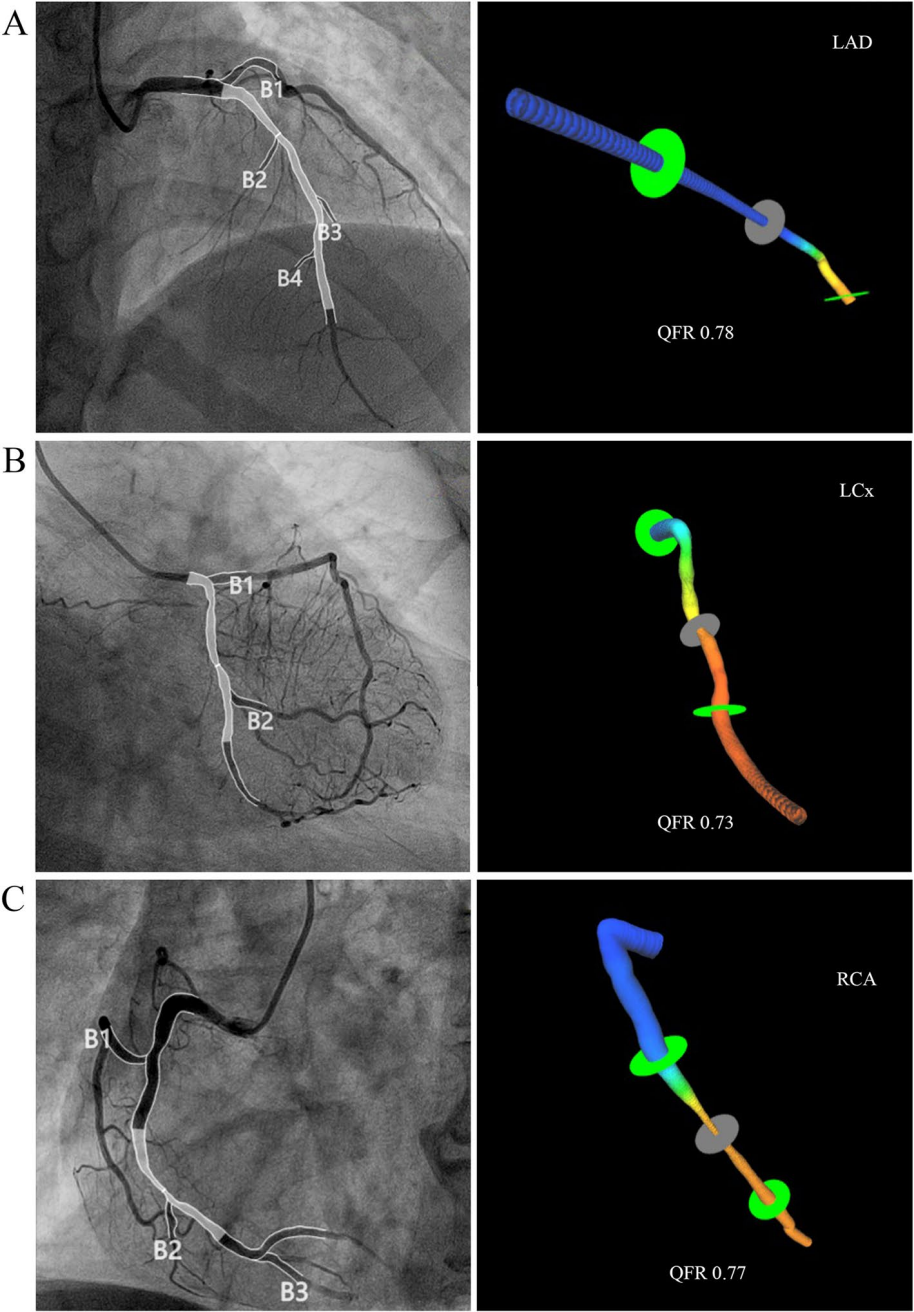
QFR analysis process. **A** The QFR of left anterior descending (LAD) was calculated as 0.78. **B** The QFR of left circumflex (LCx) was calculated as 0.73. **C** The QFR of right coronary artery (RCA) was calculated as 0.77

All patients underwent coronary angiography according to standardized protocols, and the subsequent QFR analysis was performed collaboratively by a team of four physicians. With the assistance of a skilled imaging specialist, two experienced cardiologists utilized the AngioPlus system (developed by Bodong Medical Imaging Technology Co., Ltd., Shanghai, China) to collectively evaluate the degree of coronary artery stenosis for each patient (Fig. 2). Additionally, a fourth clinical physician was assigned to meticulously validate the data. In cases where suboptimal image quality was observed, manual adjustments were made following established procedural guidelines.

Subsequently, the contrast agent flow velocity was determined using frame counting methodology derived from the coronary angiography images. A contrast agent flow model was then employed to calculate the quantitative flow ratio (QFR) values obtained from the analysis. QFR, as a quantitative indicator, effectively reflected the functional severity of coronary artery stenosis. The target vessel for further analysis was selected based on the presence of the most severe stenosis. QFR values were quantified to assess the degree of functional stenosis in the target vessel and compared accordingly. Consistent with previous research [22], a QFR value of ≤ 0.80 was considered indicative of functionally significant coronary artery stenosis.

### Statistical methods

Data analysis was conducted using SPSS software version 27.0. The normality of continuous variables was assessed using the Kolmogorov–Smirnov test. Continuous variables were reported as mean ± standard deviation (SD) or median (interquartile range), while categorical variables were presented as frequencies (percentages). The differences in continuous variables between the two groups were evaluated using an independent t-test for normally distributed variables and the Mann–Whitney U test for non-normally distributed variables. Categorical variables were compared using the chi-square test.

In order to investigate the relationship between composite parameters and QFR ≤ 0.80, the composite parameters were treated as continuous variables. To achieve normal distribution, a natural logarithm (ln) transformation was applied to larger numeric values, while an exponential transformation (e^n) was applied to smaller numeric values. The optimal cutoff value for the composite parameters associated with QFR ≤ 0.80 was determined using receiver operating characteristic (ROC) analysis. The diagnostic performance of UAR was compared to other composite markers (SII, SIRI, AISI, and MHR) in identifying QFR ≤ 0.80. The discriminatory ability of UAR and other composite markers for QFR ≤ 0.80 was assessed by calculating the area under the ROC curve. The composite parameters were dichotomized based on the optimal cutoff value determined by the ROC analysis, and both univariate and multivariate logistic regression analyses were performed to determine the correlation between the relevant composite parameters and QFR ≤ 0.80. Statistical significance was defined as a two-tailed *p*-value < 0.05.

## Results

### Comparison of general clinical data

A total of 257 suspected coronary artery disease patients were enrolled in the study, comprising 83 individuals in the QFR ≤ 0.80 group and 174 individuals in the QFR > 0.80 group. The prevalence of hypertension in the QFR ≤ 0.80 group was significantly higher than that in the QFR > 0.80 group (*p* < 0.001) (Table 1).

**Table 1:** Characteristics of study population

Variables	QFR ≤ 0.80(*n* = 83)	QFR > 0.80(*n* = 174)	*p*
Clinical Characteristics			
Male [*n* (%)]	49(59.0)	103(59.2)	0.981
Age, years, median (IQR)	62.23 ± 10.05	61.84 ± 8.74	0.750
Hypertension, *n* (%)	53(63.9)	81(46.6)	< 0.001
Previous stroke, *n* (%)	5(6.0)	9(5.2)	0.774
Diabetes, *n* (%)	21(25.3)	27(15.5)	0.086
Current smoking, *n* (%)	26(31.3)	44(25.3)	0.369
Laboratory Characteristics			
Rapid glucose, mmol/L	7.46 ± 2.70	7.09 ± 2.77	0.311
HbA1c (%)	6.66 ± 1.18	6.37 ± 0.98	0.040
TC, mmol/L	4.66 ± 1.13	4.33 ± 1.05	0.022
TG, mmol/L	2.32 ± 0.70	1.98 ± 1.29	0.079
HDL-c, mmol/L	1.14 ± 0.31	1.23 ± 0.30	0.040
LDL-c, mmol/L	2.50 ± 0.84	2.23 ± 0.70	0.007
Platelet count, 10^9^/L	216.94 ± 63.59	200.07 ± 57.41	0.034
Neutrophil count, 10^9^/L	4.63 ± 1.65	3.76 ± 1.17	< 0.001
Lymphocyte count, 10^9^/L	1.61 ± 0.60	1.45 ± 0.55	0.028
Monocyte count, 10^9^/L,	0.49 ± 0.15	0.42 ± 0.15	0.001
Creatinine, mg/dl	78.5 ± 22.17	76.7 ± 22.20	0.548
Uric acid, µmol/L	374.78 ± 90.08	328.51 ± 77.81	< 0.001
Albumin, g/L	41.15 ± 3.31	43.42 ± 3.13	< 0.001
Composite parameters			
UAR	9.19 ± 2.47	7.61 ± 1.91	< 0.001
MHR	0.46 ± 0.19	0.37 ± 0.16	< 0.001
SII	674.98 ± 332.30	571.43 ± 255.82	0.006
SIRI	1.53 ± 0.83	1.29 ± 1.10	0.047
AISI	340.22 ± 242.10	243.97 ± 151.97	< 0.001
QFR Characteristics			
Target vessel, *n* (%)			
LAD	56(67.5)	96(55.2)	0.077
LCX	13(15.7)	43(24.7)	0.109
RCA	14(16.9)	35(20.1)	0.612
DS(%)	59.04 ± 12.97	29.72 ± 10.61	< 0.001
AS(%)	81.37 ± 11.89	49.46 ± 14.70	< 0.001
Microcirculatory Resistance, mmHg*s/m	146.14 ± 72.14	259.79 ± 53.21	< 0.001

Values are mean ± SD, n (%), or median (interquartile range) unless otherwise stated

*HbA1c* Glycosylated Hemoglobin, Type A1C, *TC* total cholesterol, *HDL-C* high-density lipoprotein cholesterol, *LDL-C* low-density lipoprotein cholesterol, *MHR* monocyte/high-density lipoprotein cholesterol ratio, *UAR* uric acid/albumin ratio, *SII* systemic immune-inflammation index, *SIRI* systemic inflammatory response index, *AISI* aggregate index of systemic inflammation

Significant differences in laboratory parameters were observed between the QFR ≤ 0.80 and QFR > 0.80 groups, including glycated hemoglobin (*p* = 0.040), total cholesterol (*p* = 0.022), low-density lipoprotein cholesterol (*p* = 0.007), platelet count (*p* = 0.034), neutrophil count (*p* < 0.001), lymphocyte count (*p* = 0.028), monocyte count (*p* = 0.001), uric acid (*p* = 0.001), and albumin (*p* < 0.001).

**Fig. 3:**
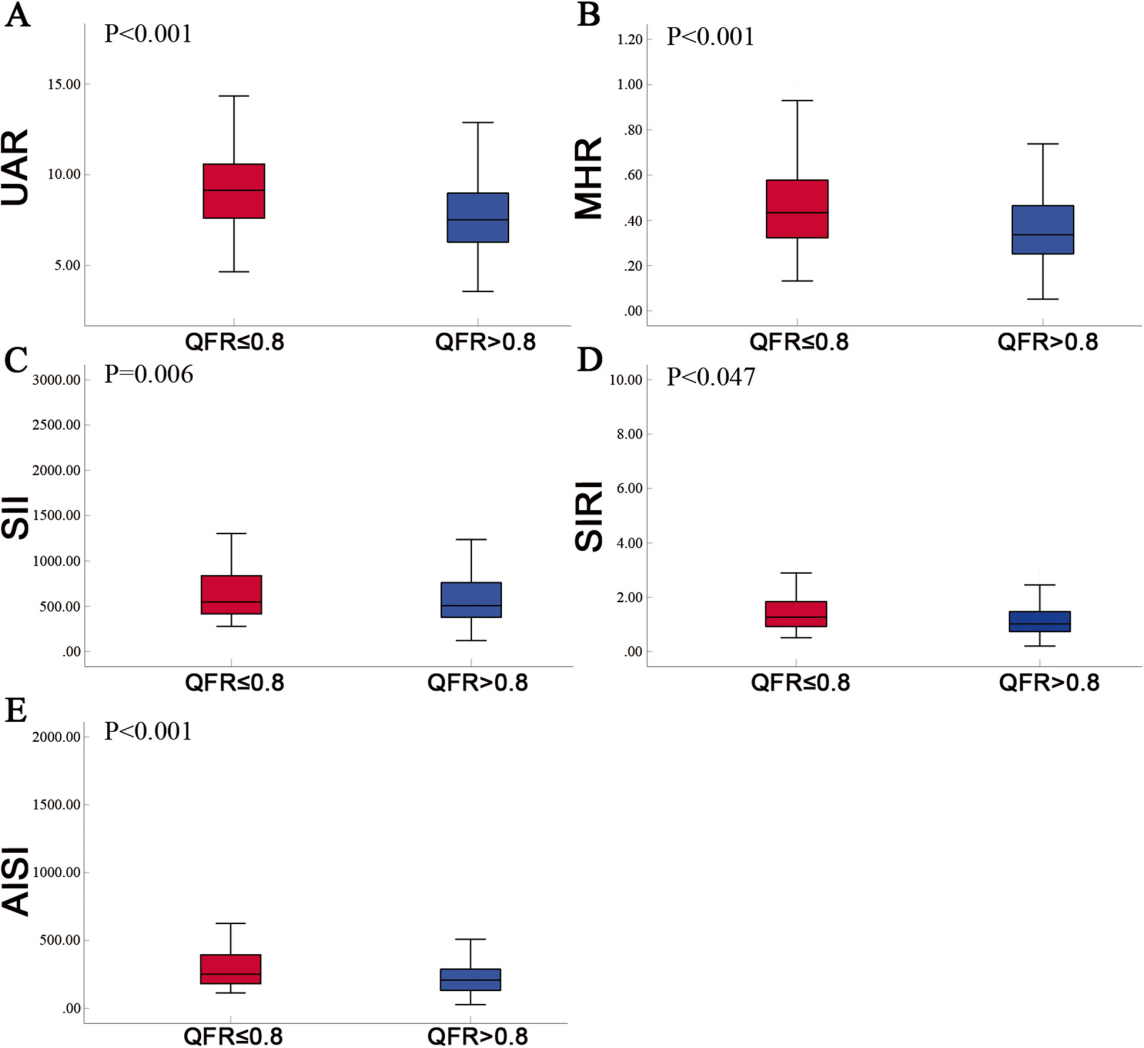
Levels of Composite parameters between QFR > 0.8 and QFR ≤ 0.8. UAR: uric acid/albumin ratio; MHR: monocyte/high-density lipoprotein cholesterol ratio; SII: systemic immune-inflammation index; SIRI: systemic inflammatory response index; AISI: aggregate index of systemic inflammation. A *p*-value < 0.05 was considered a statistically significant difference between the two groups

Regarding the composite parameters, statistically significant differences were observed between the QFR ≤ 0.80 and QFR > 0.80 groups for UAR (9.19 ± 2.47 vs 7.61 ± 1.91; *p* < 0.001), MHR (0.46 ± 0.19 vs 0.37 ± 0.16; *p* < 0.001), SII (674.98 ± 332.30 vs 571.43 ± 255.82; *p* = 0.006), SIRI (1.53 ± 0.83 vs 1.29 ± 1.10; *p* = 0.047), and AISI (340.22 ± 242.10 vs 243.97 ± 151.97; *p* < 0.001). Notably, UAR, MHR, and AISI demonstrated a stronger correlation with QFR ≤ 0.80 (Fig. 3).

### ROC curve analysis

**Fig. 4:**
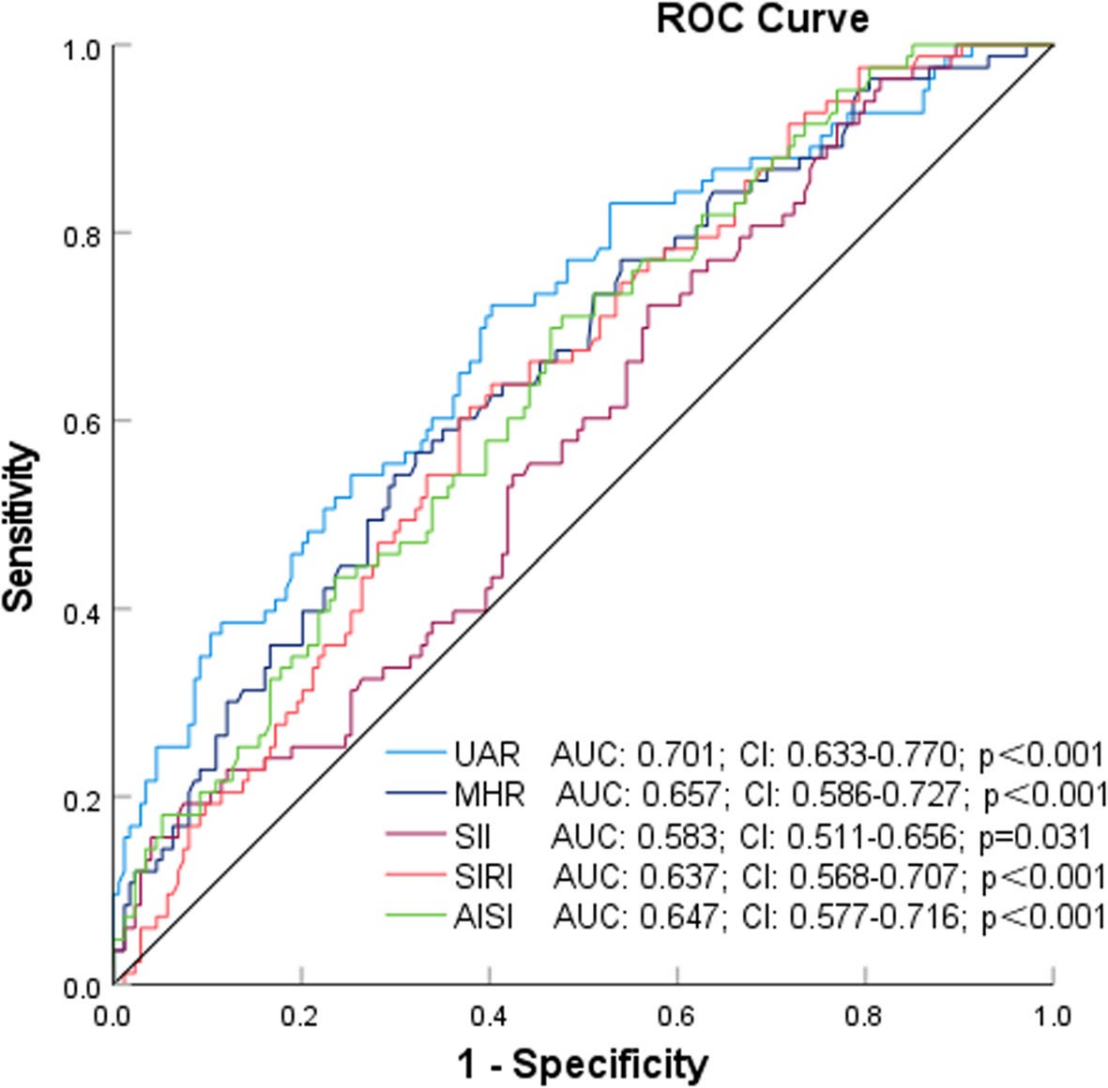
Receiver Operating Characteristic Curves of UAR, MHR, SII, AISI, and SIRI as Variables With Coronary Artery Stenosis

**Fig. 5:**
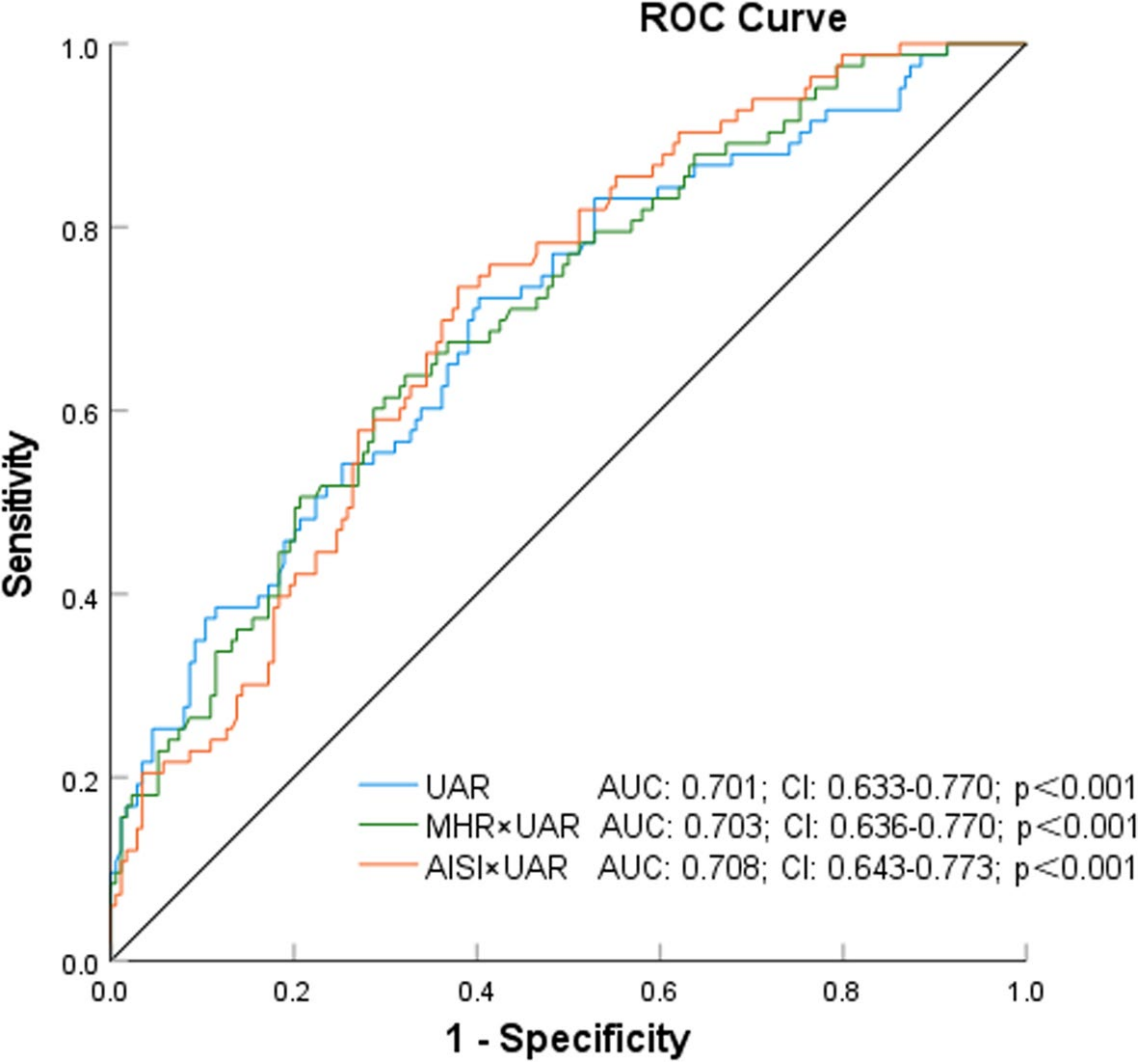
Receiver Operating Characteristic Curves of UAR, UAR × AISI and UAR × SIRI as Variables With Coronary Artery Stenosis

**Fig. 6:**
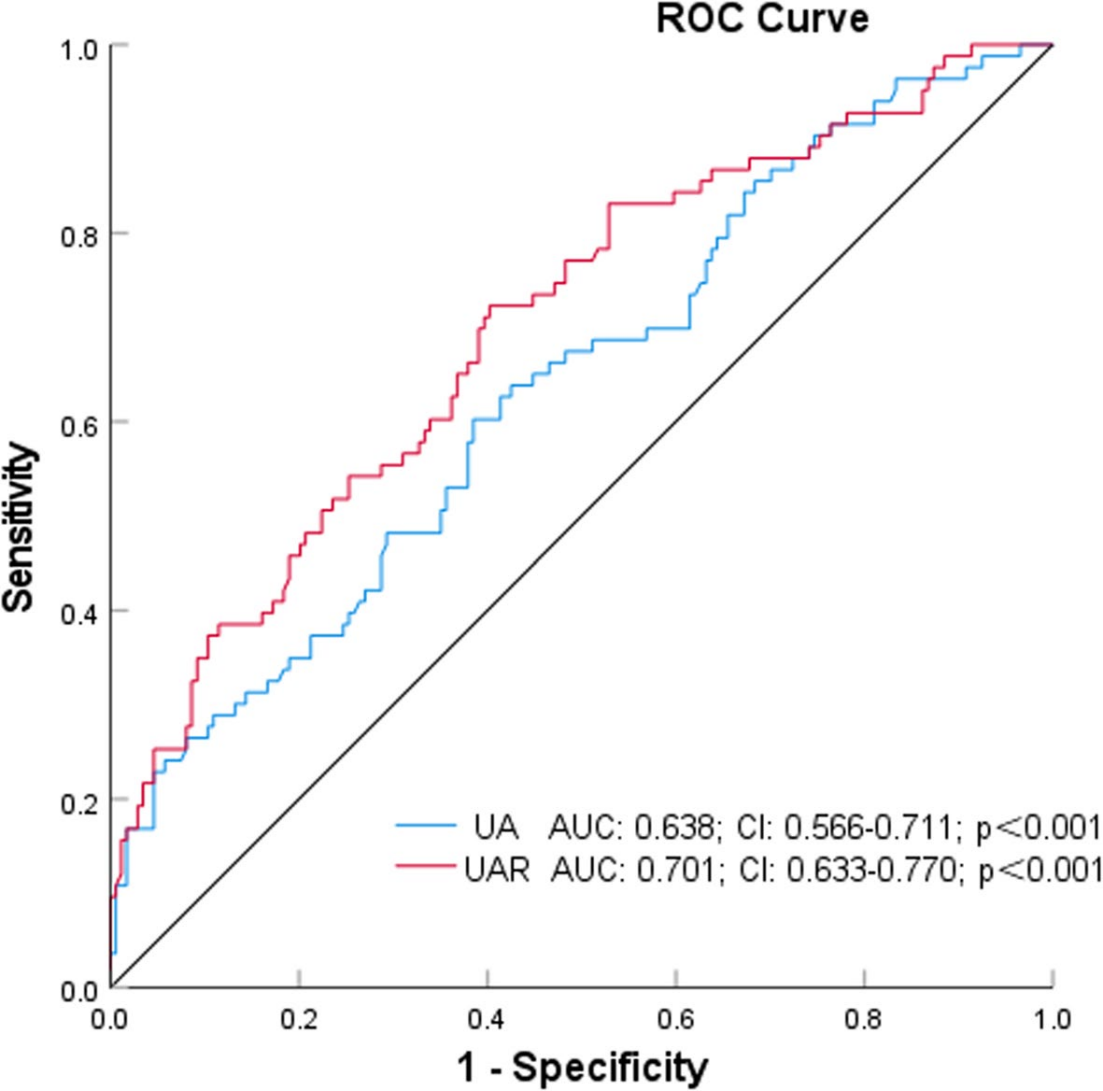
Receiver Operating Characteristic Curves of UA and UAR as Variables With Coronary Artery Stenosis

The analysis of the ROC curve revealed that the area under the curve (AUC) for UAR was determined to be 0.701 (95% confidence interval [CI]: 0.633–0.770; *p* < 0.001). In comparison to UA, UAR continued to demonstrate superior performance (Figs. 4, 5 and 6). Furthermore, the combined parameters MHR × UAR and AISI × UAR did not exhibit a significant enhancement in AUC when compared to UAR alone (Table 2).

**Table 2:** Comparison of AUC for different indicators

Indicator	AUC	se	*P* value	95% CI		Cutoff value	Sensitivity	Specificity
				Lower	Upper			
UAR	0.701	0.035	0.000	0.633	0.770	7.92	0.72	0.60
UA	0.638	0.037	0.000	0.566	0.711	342.95	0.60	0.61
MHR	0.657	0.036	0.000	0.586	0.727	0.40	0.57	0.68
SII	0.583	0.037	0.031	0.511	0.656	468.26	0.72	0.43
SIRI	0.637	0.035	0.000	0.568	0.707	1.14	0.64	0.60
AISI	0.647	0.035	0.000	0.577	0.716	213.73	0.71	0.52
MHR × UAR	0.703	0.034	0.000	0.636	0.770	3.30	0.64	0.68
AISI × UAR	0.708	0.034	0.000	0.643	0.773	1791.59	0.73	0.62

*UAR* uric acid/albumin ratio, *UA* uric acid, *MHR* monocyte/high-density lipoprotein cholesterol ratio, *SII* systemic mmune-inflammation index, *SIRI* systemic inflammatory response index, *AISI* aggregate index of systemic inflammation

### Univariate and multivariate logistic regression analysis

Through the analysis of the ROC curve, we determined the optimal cutoff values for five composite parameters, namely UAR, MHR, SII, SIRI, and AISI. These cutoff values were used to transform the continuous variables into binary variables. To address multicollinearity issues, we selected Hypertension, Hemoglobin A1c (HbA1c), Low-Density Lipoprotein Cholesterol (LDL-c), MHR, SII, SIRI, AISI, and UAR as independent variables for logistic regression analysis (Table 3). Our findings revealed a significant association between elevated levels of UAR (Odds Ratio [OR]: 3.876, 95%CI: 2.196–6.841, *p* < 0.001), MHR (OR: 2.751, 95% CI: 1.606–4.712, *p* < 0.001), SII (OR: 1.976, 95% CI: 1.121–3.483, *p* < 0.018), SIRI (OR: 2.444, 95% CI: 1.425–4.193, *p* < 0.001), and AISI (OR: 2.695, 95% CI: 1.539–4.719, *p* < 0.001) with a significantly increased risk of QFR ≤ 0.80 (refer to Table 2). Furthermore, in the multivariate analysis, elevated levels of UAR (OR: 3.085, 95% CI: 1.722–5.527, *p* < 0.001) and AISI (OR: 2.269, 95% CI: 1.256–4.099, *p* = 0.007) remained significantly associated with the risk of QFR ≤ 0.80.

**Table 3:** Univariate and multivariate logistic regression analysis of predictors of UAR, MHR, SII, SIRI, and AISI evaluated as categorical variables with coronary artery stenosis

	Univariate analysis		Multivariate analysis	
Variable	OR (95% CI)	*p*	OR (95% CI)	*p*
Hypertension, n (%)	2.028(1.185–3.473)	0.010	1.859 (1.044–3.309)	0.035
HbA1c (%)	1.278(1.006–1.624)	0.045	0.967 (0.693–1.350)	0.845
LDL-c, mmol/L	1.607(1.132–2.282)	0.008	1.622 (1.113–2.363)	0.012
UAR > 9.72	3.876(2.196–6.841)	< 0.001	3.085 (1.722–5.527)	< 0.001
MHR > 0.40	2.751(1.606–4.712)	< 0.001	1.506 (0.809–2.805)	0.197
SII > 468.26	1.976(1.121–3.483)	0.018	1.298 (0.569–2.959)	0.536
AISI > 213.73	2.695(1.539–4.719)	0.001	2.269 (1.256–4.099)	0.007
SIRI > 1.14	2.444(1.425–4.193)	0.001	1.166 (0.541–2.515)	0.695

*LDL-C* low-density lipoprotein cholesterol, *UAR* uric acid/albumin ratio, *MHR* monocyte/high-density lipoprotein cholesterol ratio, *SII* systemic immune-inflammation index, *SIRI* systemic inflammatory response index, *AISI* aggregate index of systemic inflammation

When considering these variables as continuous predictors, univariate analysis demonstrated a significant association between UAR (OR: 1.412, 95% CI: 1.231–1.620, *p* < 0.001), e^MHR (OR: 1.394, 95% CI: 1.151–1.687, *p* < 0.001), lnSII (OR: 1.001, 95% CI: 1.000–1.002, *p* = 0.008), lnAISI (OR: 2.695, 95% CI: 1.539–4.719, *p* = 0.001), and QFR ≤ 0.80. In the multivariable analysis, UAR (OR: 1.373, 95% CI: 1.187–1.587, *p* < 0.001) and AISI (OR: 2.217, 95% CI: 1.309–3.757, *p* < 0.001) remained significantly associated with QFR ≤ 0.80 (refer to Table 4).

**Table 4:** Univariable and multivariable logistic regression analysis of associations of UAR, MHR, SII, SIRI, and AISI evaluated as continuous variables with coronary artery stenosis

	Univariate analysis		Multivariate analysis	
Variable	OR (95% CI)	*p*	OR (95% CI)	*p*
Hypertension, *n* (%)	2.028(1.185–3.473)	0.010	1.851 (1.021–3.355)	0.042
HbA1c (%)	1.278(1.006–1.624)	0.045	1.153 (0.884–1.503)	0.292
LDL-c, mmol/L	1.607(1.132–2.282)	0.008	1.674 (1.137–2.464)	0.009
UAR	1.412(1.231–1.620)	< 0.001	1.373 (1.187–1.587)	< 0.001
e^MHR	1.394(1.151–1.687)	< 0.001	1.602 (0.470–5.461)	0.451
lnSII	1.001(1.000–1.002)	0.008	0.778 (0.162–3.742)	0.754
lnAISI	2.695(1.539–4.719)	0.001	2.217 (1.309–3.757)	0.003
SIRI	1.279(0.997–1.674)	0.073		

*LDL-C* low-density lipoprotein cholesterol, *UAR* uric acid/albumin ratio, *MHR* monocyte/high-density lipoprotein cholesterol ratio, *SII* systemic immune-inflammation index, *SIRI* systemic inflammatory response index, *AISI* aggregate index of systemic inflammation

## Discussion

The results of this study demonstrate a significant correlation between UAR), MHR, SII, SIRI, AISI, and the severity of coronary artery functional stenosis assessed through QFR. Given that QFR is a relatively new technology, there is limited literature available on the relationship between composite parameters in the blood and QFR-based coronary artery functional assessment. Previous research has indicated the crucial role of inflammation and oxidative stress in the pathogenesis of atherosclerosis and vulnerable plaque formation [3, 4]. Conventional inflammatory biomarkers, such as complete blood cell count and C-reactive protein, have been used for acute and long-term cardiovascular risk assessment [23]. Increasingly, studies have focused on the prognostic relationship between calculated composite parameters from blood samples and cardiovascular diseases. These composite parameters often consist of blood cell counts and biochemical parameters, as seen in this study with MHR, SII, SIRI, AISI, and UAR. These parameters are not only easily obtainable and cost-effective but also reflect the severity of systemic inflammation. Research has suggested that SII and SIRI are associated with the extent of coronary artery disease in patients with atherosclerotic heart disease [8, 24].

The elevation of SII and SIRI reflects an increase in platelets, neutrophils, and monocytes, accompanied by a decrease in lymphocytes. This pattern suggests the presence of non-specific inflammation and an adaptive immune response, contributing to the progression of cellular-level damage [25]. Consistent with previous findings, Yang et al. [26] also reported a potential association between SIRI, AISI, and coronary artery stenosis. However, in our study, when considering SIRI as a continuous variable, its correlation with QFR ≤ 0.80 weakened. This observation may be attributed to the similar composition of variables (SII, SIRI, and AISI) in our study. In contrast, we found that SII and AISI showed a significant correlation with QFR ≤ 0.80, further supporting the previous research findings. Previous studies have suggested that Monocyte-to-High-Density Lipoprotein Cholesterol Ratio (MHR) levels could serve as a potential predictor of the severity of coronary artery lesions [27]. High-density lipoprotein plays a crucial role in inhibiting inflammatory signaling within macrophages and other cells. However, in patients, oxidative modifications can impair the functionality of high-density lipoprotein, leading to a pro-inflammatory state [28]. In line with these previous findings, our study demonstrated a significant correlation between MHR and QFR ≤ 0.80, indicating its potential as a relevant marker. Overall, our study provides valuable insights into the relationship between composite parameters in the blood and the severity of coronary artery functional stenosis assessed through QFR. The elevated levels of SII, SIRI, and MHR suggest underlying systemic inflammation and immune response, which contribute to the progression of coronary artery disease. These findings contribute to a better understanding of the pathophysiological mechanisms and potential prognostic indicators in cardiovascular diseases.

The UAR has been found to be significantly associated with the severity of arterial atherosclerosis [29]. As a novel inflammatory biomarker, UAR has demonstrated independent and reliable predictive capabilities in determining the extent of coronary artery stenosis in patients with Non-ST-segment elevation myocardial infarction (NSTEMI) [30]. However, the precise mechanisms underlying the involvement of UAR in the development and severity of CAD remain incompletely understood. Elevated levels of uric acid UA have been implicated in enhanced oxidative stress, inhibition of the nitric oxide system, and activation of the renin-angiotensin system [31–33]. Moreover, higher serum uric acid levels have been associated with increased vulnerability of coronary artery plaques [34]. Additionally, albumin exhibits a range of biological functions, including antioxidant and anti-inflammatory properties [35]. Therefore, it is postulated that the impact of UAR on CAD involves multiple pathways and factors, suggesting its potential as a valuable tool for risk stratification in CAD. In conclusion, UAR has emerged as a promising marker associated with the severity of arterial atherosclerosis and holds independent predictive value for assessing coronary artery stenosis in NSTEMI patients. Its involvement in CAD is likely multifaceted and encompasses various mechanistic aspects. Further investigations are warranted to comprehensively elucidate the underlying mechanisms and evaluate the clinical utility of UAR in risk stratification for CAD.

The CTA and FFR are commonly utilized modalities for assessing the severity of coronary artery stenosis. A study [36] aimed to investigate and compare the predictive capabilities of SII, Neutrophil-to-Lymphocyte Ratio (NLR), and Platelet-to-Lymphocyte Ratio (PLR) in predicting hemodynamically significant coronary artery stenosis as determined by FFR. The study concluded that SII levels demonstrated superior predictive ability compared to NLR and PLR in determining the hemodynamic significance of coronary artery obstruction. In recent years, the rapid development of artificial intelligence has introduced a novel possibility in coronary artery physiology assessment through the introduction of QFR. Several recent studies have shown that QFR-guided strategies have improved clinical efficacy at 1-year and 2-year follow-ups [22, 33, 37–39]. Furthermore, international expert consensus has acknowledged the clinical evidence supporting image-based coronary physiological evaluation, establishing QFR as the "new standard" for international coronary physiology assessment [40]. Liu et al. [41] utilized QFR to evaluate the relationship between various immune cell cytokines and coronary artery physiological function. The study revealed that a combination of IL-6, IL-10, and CD4 + T lymphocytes outperformed individual biomarkers in predicting functional coronary artery stenosis. Combining immune-inflammatory biomarkers demonstrated high predictive value for significant functional and anatomical coronary artery stenosis. Additionally, there have been studies comparing the diagnostic performance of contrast-based Quantitative Flow Ratio (cQFR) and fixed-flow-based Quantitative Flow Ratio (fQFR) with the Resting Full-cycle Ratio (RFR) using FFR as the reference standard and found that contrast flow (cQFR) demonstrates superior diagnostic performance compared to NHPR RFR in predicting the functional significance of coronary stenoses based on FFR [42]. In our study, we aimed to identify new biomarkers and evaluation tools associated with the degree of coronary artery stenosis by combining UAR and AISI, both of which exhibited favorable diagnostic performance. Notably, we observed associations between MHR, SII, SIRI, AISI, and UAR with coronary artery functional stenosis, further supporting the value of these composite parameters as assessment tools. These findings provide novel insights and a robust theoretical foundation for the early diagnosis and management of coronary artery disease.

### Limitations

Our study has several limitations that should be acknowledged. Firstly, it is important to note that our study design was retrospective and cross-sectional in nature. The utilization of QFR technology in coronary angiography images demands a high level of technical expertise and adherence to standardized protocols to ensure accurate data collection. Consequently, the sample size in our study was relatively small. To address these limitations and further explore the relationships between the parameters under investigation, it would be valuable to extend the study duration and collaborate with multiple centers to conduct large-scale, multicenter cohort studies. Secondly, it is worth mentioning that our study did not include a comparison between these parameters and patients with multi-vessel coronary artery disease. Additionally, we were unable to compare the severity of vascular lesions within the same patient due to the limitations of QFR evaluation in certain coronary vessels. These factors contribute to a gap in our understanding of the associations between these parameters and more complex coronary artery disease presentations. This study did not include complex lesions such as coronary ostial disease, severe vessel tortuosity, and diffuse long lesions. Therefore, the findings of this study may not represent patients with more severe and complex coronary artery disease. Thirdly, it is important to acknowledge that our study did not incorporate C-reactive protein (CRP) and its related composite inflammatory parameters, despite their potential relevance. A previous study [43] highlighted the association between neutrophil count, SII, CRP, albumin, and the CRP-to-albumin ratio (CAR) with the occurrence of microvascular angina (MVA) in univariate analysis. Future research should consider the inclusion of these inflammatory markers to provide a more comprehensive assessment of their impact on coronary artery disease. Another limitation of this study is that although it analyzed the value of serological markers in assessing the disease, it did not incorporate treatment regimens to analyze patient prognostic outcomes. This calls for further clinical information to be provided through cohort studies that consider treatment regimens. Lastly, it is essential to recognize that our study did not account for the potential influence of oral medications that patients may have been taking prior to admission. These medications could have introduced confounding factors that may have affected the blood markers under investigation. Future studies should consider controlling for medication history to ensure a more accurate evaluation of the parameters of interest.

## Conclusions

When evaluating the degree of functional stenosis in patients with CAD, the UAR has emerged as a more effective independent indicator compared to composite parameters derived from blood laboratory markers. Consequently, in clinical practice, UAR holds promise in predicting the extent of functional stenosis in CAD patients and facilitating risk stratification for CAD. Notably, the AISI has been identified as a representative inflammatory biomarker within the complete blood cell count, capable of influencing the severity of coronary artery physiological impairment.

## Data Availability

No datasets were generated or analysed during the current study.
